# Integration of Gene Dosage and Gene Expression in Non-Small Cell Lung Cancer, Identification of HSP90 as Potential Target

**DOI:** 10.1371/journal.pone.0001722

**Published:** 2008-03-05

**Authors:** Mariëlle I. Gallegos Ruiz, Karijn Floor, Paul Roepman, José A. Rodriguez, Gerrit A. Meijer, Wolter J. Mooi, Ewa Jassem, Jacek Niklinski, Thomas Muley, Nico van Zandwijk, Egbert F. Smit, Kristin Beebe, Len Neckers, Bauke Ylstra, Giuseppe Giaccone

**Affiliations:** 1 Department of Medical Oncology, Vrije Universiteit Medisch Centrum, Amsterdam, The Netherlands; 2 Agendia BV, Amsterdam, The Netherlands; 3 Department of Pathology, Vrije Universiteit Medisch Centrum, Amsterdam, The Netherlands; 4 University of Gdansk, Gdansk, Poland; 5 University of Bialystok, Bialystok, Poland; 6 Thoraxklinik Heidelberg, University of Heidelberg, Heidelberg, Germany; 7 Netherlands Cancer Institute, Amsterdam, The Netherlands; 8 Department of Pulmonary Diseases, Vrije Universiteit Medisch Centrum, Amsterdam, The Netherlands; 9 Urologic Oncology Branch, Center for Cancer Research, National Cancer Institute, Bethesda, Maryland, United States of America; Ohio State University, United States of America

## Abstract

**Background:**

Lung cancer causes approximately 1.2 million deaths per year worldwide, and non-small cell lung cancer (NSCLC) represents 85% of all lung cancers. Understanding the molecular events in non-small cell lung cancer (NSCLC) is essential to improve early diagnosis and treatment for this disease.

**Methodology and Principal Findings:**

In an attempt to identify novel NSCLC related genes, we performed a genome-wide screening of chromosomal copy number changes affecting gene expression using microarray based comparative genomic hybridization and gene expression arrays on 32 radically resected tumor samples from stage I and II NSCLC patients. An integrative analysis tool was applied to determine whether chromosomal copy number affects gene expression. We identified a deletion on 14q32.2-33 as a common alteration in NSCLC (44%), which significantly influenced gene expression for HSP90, residing on 14q32. This deletion was correlated with better overall survival (P = 0.008), survival was also longer in patients whose tumors had low expression levels of HSP90. We extended the analysis to three independent validation sets of NSCLC patients, and confirmed low HSP90 expression to be related with longer overall survival (P = 0.003, P = 0.07 and P = 0.04). Furthermore, in vitro treatment with an HSP90 inhibitor had potent antiproliferative activity in NSCLC cell lines.

**Conclusions:**

We suggest that targeting HSP90 will have clinical impact for NSCLC patients.

## Introduction

Lung cancer is the leading cause of cancer deaths worldwide [1], and non-small cell lung cancer (NSCLC) represents 85% of lung cancers. A better understanding of the molecular events underlying the development and progression of the disease may contribute to improve clinical management of NSCLC patients. A number of genes, e.g. P53, RAS, P16 and EGFR, have been shown to be altered in NSCLC [2]. Given the heterogeneous and complex nature of this tumor type, it is likely that many genes driving NSCLC tumorigenesis have yet to be identified.

Chromosomal aberrations are thought to be critical events in human tumorigenesis, and several genomic regions frequently harboring DNA gains (3q, 5p, 7q, 8q, 11q and 16p) and losses (3p, 4q, 5q, 6q, 8p 9p and 13q, 17q) have been identified in NSCLC patients [3]. Using array based comparative genomic hybridization (aCGH) and gene expression microarrays, DNA copy number changes and gene expression can be measured throughout the whole genome of tumor cells. By combining the data from these analyses, it is possible to obtain an integrated genome wide view of gene dosage aberrations and their effect on gene expression, which might help in identifying genes important in NSCLC [4].

In the present study, we have performed an integrative analysis of chromosomal copy number and gene expression on radically resected tumor samples from 32 NSCLC patients. Two new algorithms, ‘CGH call’[5] and ‘ACE-it’[6], were applied to analyze the data. We identified a deletion on chromosome region 14q32.2-33 in 44% of NSCLC patients. This deletion was related with improved patient survival, and was associated with decreased expression of HSP90, a molecular chaperone for several oncoproteins that is being explored as a novel target in anticancer therapy. Low HSP90 expression was correlated with improved survival in the 32 NSCLC patients analyzed initially. Further analysis of three independent sets of NSCLC patients confirmed a significant association between patient survival and HSP90 expression. In addition, *in vitro* experiments show NSCLC cell lines to be extremely sensitive to the HSP90 inhibitor 17-AAG. Our data suggest and important role for HSP90 in NSCLC.

## Methods

### Patients and samples

The test set consisted of radically resected tumor specimens of 32 early stage NSCLC patients. Three patients had a survival time of less than 30 days and were considered postoperative deaths. Therefore these three patients are not included in the survival analyses. Patients had a median follow up of 86 months (range 0.4-135.5). Verbal informed consent had been obtained from all patients and handling of samples was in accordance with protocols approved by the ethical board “subcommissie voor de ethiek van het mensgebonden onderzoek” from the VU University Medical Center in Amsterdam.

The first validation set consisted of 140 radically resected NSCLC patients from the European lung cancer consortium. Patients had a median follow up of 35 months. All patients included had had no prior malignancy, pathological tumor stage 1 or 2 (T1-2), node stage 0+1 (N0-1), no distant metastasis (M0) at time of operation, and no residual disease after resection (R0). None of these patients received (neo)adjuvant chemo- or radiotherapy. The second validation consisted of 111 early stage NSCLC patients from Bild et al. [7]. The third validation set consisted of the publicly available “datasets 1 and 2” from Lu et al. [8] and contained 54 early stage NSCLC patients. A full description of patient characteristics of all four patient sets is provided in Table 1.

**Table 1:** Clinical characteristics of test and validation patient sets

Characteristic	Test set		Validation set 1		Validation set 2		Validation set 3	
	n = 32		n = 140		n = 111		n = 54	
	n	(%)	n	(%)	n	(%)	n	(%)
Gender					n/a			
Male	24	(75)	105	(75)			24	(44)
Female	8	(25)	35	(25)			29	(54)
Histology								
Adenocarcinoma	13	(41)	43	(31)	58	(52)	14	(26)
Squamous Cell Carcinoma	15	(47)	78	(56)	53	(48)	36	(67)
Large Cell Carcinoma	3	(9)	7	(5)	0	(0)	4	(7)
Others	1	(3)	12	(8)	0	(0)	0	(0)
Smoking status					n/a		n/a	
Never	0	(0)	7	(5)				
Former	16	(50)	57	(41)				
Current	11	(34)	65	(46)				
Unknown	5	(16)	11	(8)				
Tumor stage								
IA	14	(44)	25	(18)	30	(27)	47	(87)
IB	9	(28)	68	(48)	27	(24)	7	(13)
IIA	2	(6)	5	(4)	0	(0)	0	(0)
IIB	7	(22)	42	(30)	0	(0)	0	(0)
	median	[range]	median	[range]	median	[range]	median	[range]
Age at diagnosis-years	67	22–78	64	37–79	n/a		66	48–81
Overall Survival-months	34	0.4–135.5	35	0.5–156	31	1–87.5	50	2–81

Percentages that do not reach 100% indicate missing data; n/a = information not publicly available

### Isolation of genomic DNA and array Comparative Genomic Hybridization

Cryo-sections of frozen tissue samples, flanking the sections used for RNA and DNA isolation, were verified by the study pathologist (WM) to contain at least 50% of tumor cells. Genomic DNA was extracted from each sample using Trizol following manufacturer instructions (Life Technologies, Breda, The Netherlands). DNA labeling and hybridization on CGH 30K oligonucleotide microarrays was performed as described by van den IJssel et al [9].

### RNA isolation and gene expression micro arrays

RNA isolation and cDNA labeling followed standard protocols. Hybridization was performed on Agilent platform according to standard procedures described by the manufacturer and elsewhere [10].

### Data analysis

For array CGH, spot analysis and quality control were performed using BlueFuse version 3.2 (BlueGenome, Cambridge, UK). Breakpoints, gains, losses and amplifications were detected using the algorithm CGH call. This algorithm converts raw log2ratios to absolute measures of “loss”, “normal”, “gain” or “amplification” by applying a segmentation algorithm combined with a probability mixture model [11]. In order to statistically test whether gene expression was affected by gene dosage we applied an array CGH expression integration tool, ACE-it[6], in which the called array CGH and normalized log10 ratios for expression arrays were used as input data. ACE-it uses the one-sided Wilcoxon rank sum statistics to test which chromosomal copy number aberrations recurrently affect RNA expression. Calculated p-values are adjusted for multiple testing using the Benjamini Hochberg method [12]. ACE-it only tests genes that meet the criteria of contamination and balance, which are controlled through a threshold on the number of samples. Here the threshold was set at a fixed default setting of 9 samples, meaning that only those chromosomal positions were taken into account that had at least 9 samples in one CGH calling status and no more than 9 in the other status. The entire array CGH data set of the test series (n = 32) is available at the GEO database (http://www.ncbi.nlm.nih.gov/projects/geo, accession number GSE7878). The gene expression data of both the test set (n = 32) and validation set 1 (n = 140) for the 359 genes identified by ACE-it is available at the Array Express database (www.ebi.ac.uk/aerep/login, accession number Array design: A-MEXP-749, Experiment data: E-TABM-270).

Gene expression data of validation set 2 was obtained using Affymetrix Hu133plus2 chips (GEO accession number GSE3141). The mean value of the MAS5 calculated signal intensities of four probesets detecting HSP90AA1 was used in our calculations.

The third validation set contained gene expression data from Affymetrix Hu95 and Hu133 chips (GEO accession number GSE6253). The Hu95 chip contained one probeset detecting HSP90AA1 and the Hu133 chip contained four probesets, of which the mean value of the RMA calculated signal intensities was used in our calculations.

### Statistics

An univariate cox regression analysis was performed to investigate relation of gene expression values with survival time. Survival curves were constructed using the Kaplan Meier method and differences in overall were evaluated using the log-rank test. In the test set, three patients with less than 30 days survival time were excluded from the survival analysis, as their death was considered surgical mortality. To determine the independent effects of HSP90 expression, histologic subtype, tumor stage, age and gender, a multivariate cox regression analysis was performed. A P value of less than 0.05 was considered statistically significant.

### Multiplex Ligation dependent Probe Amplification (MLPA)

For Multiplex Ligation dependent Probe Amplification (MLPA), the subtelomere probe set P070 (MRC Holland, Amsterdam, The Netherlands) containing a probe located within the band 14q32.33 (region 104874216 to 105070384) was used. MLPA was performed according to manufacturer's instructions using 100ng DNA as input. DNA isolated from blood of a pool of healthy donors was used as reference sample. Probe signals were normalized by dividing the peak area of chromosome 14q by the peak area of chromosome 14p. MLPA generated 14q/14p ratios are plotted against the mean normalized log2ratios of the oligos in area 104787271-105071522 from the array (total of 11 oligos). This region covers the region of the MLPA probe.

### Quantitative RT-PCR

In order to validate expression values obtained via gene expression arrays, we performed quantitative real-time PCR using Taqman® technology and the ABI PRISM 7500 Sequence Detection System instrument equipped with the SDS version 1.3.0 software (Applied Biosystems, Foster City, CA, USA). Forward and reverse primers and probes were designed and produced by Applied Biosystems for HSP90AA1 (Hs00743767_sH), and for the endogenous control gene GUSB (Hs00939626_mi). PCR was carried out in a 25-µl reaction volume that contained 50 ng of cDNA, 1× TaqMan Universal PCR Master Mix, and the primer and probe sets for HSP90AA1 and GUSB. Each sample was analyzed in duplicate, and the average threshold cycle (Ct) values of each sample for GUSB, were subtracted from the average Ct values for HSP90AA1. Taqman-generated ΔCt values were log transformed to expression values and plotted against the Δlog2ratios between GUSB and HSP90AA1 from the expression array.

### Cell growth inhibition studies

Growth inhibition following *in vitro* exposure to the clinically used Hsp90 inhibitor 17-AAG (InvivoGen, San Diego, CA) was examined in 4 EGFR wild type NSCLC cell lines (H460, H157, H441 and A549, obtained from Drs. P. Dennis and F. Kaye, NCI, Bethesda, MD). Briefly, 10^5^ cells were seeded in 6-well tissue culture plates (Sigma-Aldrich, St. Louis, MO), allowed to adhere, and then continuously exposed to various concentrations of 17-AAG (0, 10, 30, 100, 300, 1000 nM) for 24, 48, or 72 hours. At each time point, cells were detached from the wells, incubated with trypan blue, and viable (trypan blue-excluding) cell number was determined in triplicate using a hemacytometer. The mean cell number with standard error bars is shown at each time point. In addition, the IC_50_ (drug concentration at which 50% growth inhibition is obtained) of 17-AAG at 72 hours was determined for each cell line.

## Results

### Gene dosage-related gene expression changes in NSCLC

**Figure 1:**
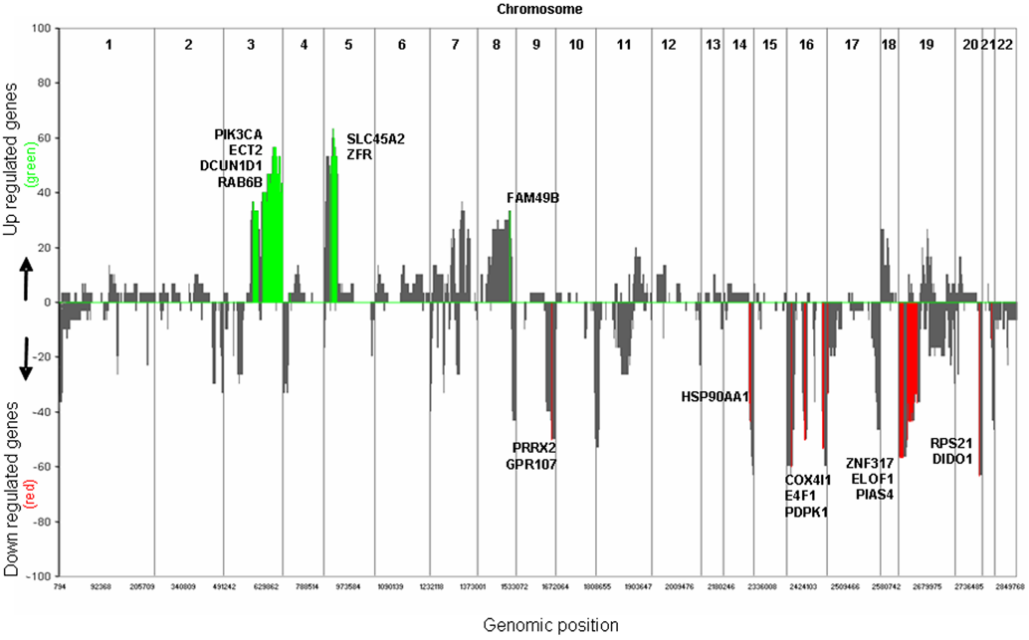
Percentage of called gains and losses and their effect on gene expression in 32 NSCLC patients. Summary plot for called gains and losses in 32 resected NSCLC patients with DNA copy number changes indicated in grey. Positive values indicate the percentage of samples found with a gain. Negative values indicate the percentage of samples harboring a loss at the specified chromosome location. Genes in specified regions affected by copy number gain are indicated in green and genes affected by copy number loss are indicated in red. A selection of affected genes is indicated. The full list of 359 affected transcripts can be found in supplementary table S1.

Chromosomal aberrations were abundant in the 32 NSCLC patients analyzed. In order to identify breakpoints of gains and losses we applied the algorithm CGH call [11]. In Table 2 the chromosomal regions in which gains or losses were present in at least 20% of patients are listed. The statistical tool ACE-it [6] was used to determine whether gene copy number affected gene expression. A total of 359 transcripts turned out to be significantly affected by copy number. In Figure 1 the areas of affected genes are indicated for 32 NSCLC patients, shown in green (gained regions) or red (lost regions).

**Table 2:** Chromosomal regions with gains and losses present in >20% of NSCLC patients analyzed

Gains				
Chromosome	Position		Band	
	Start	End	Start	End
3	95285925	126749042	3q11.2	3q21.2
3	130358402	199253846	3q21.3	3q29
5	237445	50384805	5p15.33	5q11.1
7	8160729	26912147	7p21.3	7p15.2
7	102223012	123576527	7q22.1	7q31.32
7	129933159	137018487	7q32.2	7q33
8	56600324	134124595	8q12.1	8q24.22
18	201402	8190420	18p11.32	18p11.23

Losses				
Chromosome	Position		Band	
	Start	End	Start	End
1	794287	6218763	1p36.33	1p36.31
2	218493156	220321649	2q35	2q35
2	239090261	242663692	2q37.3	2q37.3
3	46687093	52531946	3p21.31	3p21.1
4	76846	9755709	4p16.3	4p16.1
5	175887337	176879711	5q35.2	5q35.3
7	249767	2725138	7p22.3	7p22.3
7	44521369	44915245	7p13	7p13
8	142432747	146195957	8q24.3	8q24.3
9	127235226	138292321	9q33.3	9q34.3
10	132822632	135257796	10q26.3	10q26.3
11	189965	3616421	11p15.5	11p15.4
11	63500334	67523686	11q13.1	11q13.2
12	131354356	131915096	12q24.33	12q24.33
14	99160871	106031435	14q32.2	14q32.33
16	37498	4794625	16p13.3	16p13.3
16	27698990	31630451	16p11.2	16p11.2
16	66028796	66259430	16q22.1	16q22.1
16	82507053	88765762	16q24.1	16q24.3
17	33712	1922929	17p13.3	17p13.3
17	73628337	78645922	17q25.3	17q25.3
19	239096	19651642	19p13.3	19p13.11
19	54738209	56174941	19q13.33	19q13.41
19	60553920	60910750	19q13.42	19q13.42
19	63553599	63784634	19q13.43	19q13.43
20	3156113	3846872	20p13	20p13
20	59726290	62367223	20q13.33	20q13.33
21	42368792	46898780	21q22.3	21q22.3
22	36290852	36537184	22q13.1	22q13.1

An univariate cox regression survival analysis was performed for expression of all 359 genes that were identified to be influenced by copy number (see supplementary table S1). After multiple testing correction (using the Benjamini Hochberg method) none of 359 genes remained significant for survival on this small patient set (n = 32). The top list of genes correlated with survival (ranked on raw p-values) contained mainly genes located on chromosomes 3 and 5 gained regions. We also observed one gene in the top-20 list, HSP90AA1, located on chromosome 14. The HSP90AA1 gene (generally referred to as HSP90), located on 14q32.2, was the only gene in this region with significantly reduced expression in patients affected by loss of this region (P = 0.05). These observations prompted us to investigate this locus in more detail.

### Genomic aberrations on 14q and HSP90AA1 gene expression correlation with survival

**Figure 2:**
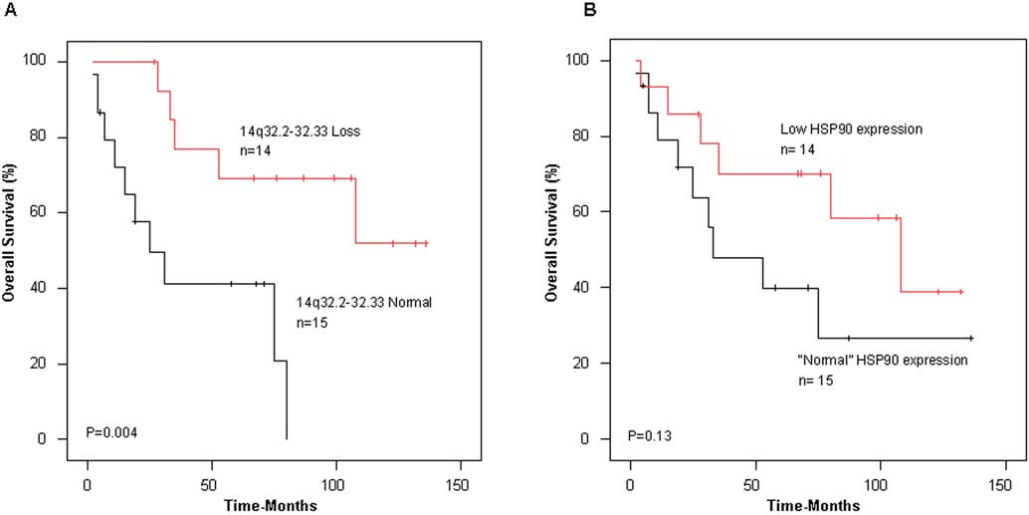
Loss of 14q32.2-32.33 chromosomal region and HSP90 expression in relation with survival. (A) Kaplan-Meier curves for overall survival are shown for 29 patients in relation to gene dosage in chromosome region 14q32.33. (B) Overall survival for 29 patients in relation to HSP90 expression. Low expression was defined as expression lower than the median of the total 32 samples, “normal” expression was defined as higher than the median of 32 samples. Three of 32 patients included in the analysis of gene dosage and expression were excluded from the survival analysis because of a survival time of less than 30 days.

We investigated the correlation of the recurrent tight deletion at region 14q32.2-33, with survival. Interestingly, patients in whom this region was deleted had an improved overall survival (OS) compared to patients harboring a normal gene dosage at this locus (5 year OS 69% vs. 41%, P = 0.004-Figure 2A).

In order to investigate HSP90 expression in relation with survival, we divided the 32 patients into two groups based on their survival status two years after tumor resection. About half of the patients were categorized as “high-risk” and half as “low-risk”. To identify whether both risk groups could be discriminated using gene expression of HSP90, we constructed Kaplan Meier survival estimates for two equally large groups with “low” and “normal”, based on the median HSP90 expression. Low expression of HSP90 was associated with better overall survival (5 year OS 70% vs. 40%, P = 0.13–Figure 2B) although differences between the two groups were initially not significant due probably to a combination of a low magnitude of the difference and a low number of patients analyzed.

### Technical validation of 14q deletion and HSP90 expression

To validate the deletion observed in region 14q32.2-33 we performed multiplex ligation dependent probe amplification [13] (MLPA) analysis using a subtelomere probeset containing a probe in region 14q32.33. The correlation coefficient (R^2^) between the loss of this area detected by array CGH and by MLPA was 0.439.

To validate the HSP90 expression data obtained with microarrays we performed quantitative RT PCR using the Taqman® technology. A good correlation between the expression of HSP90 measured with the two different techniques was observed (R^2^ = 0.6431).

### Validation of HSP90 expression and relation with survival in independent patient series

**Figure 3:**
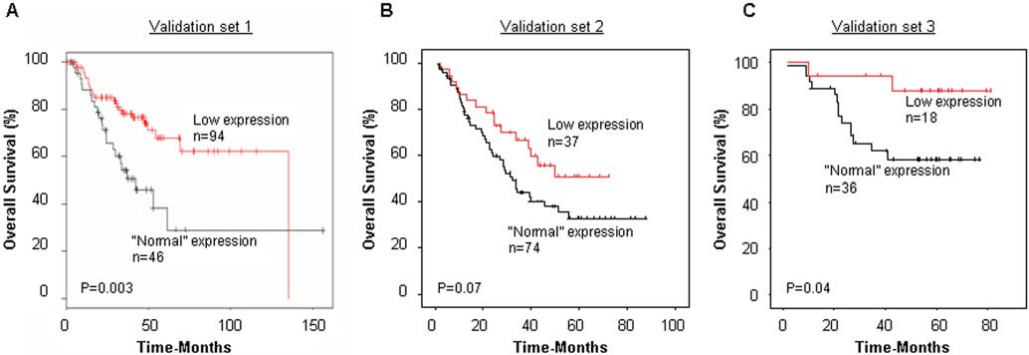
HSP90 expression and survival in three validation sets of NSCLC patients. Overall survival for (A) 140 patients with NSCLC, validation set 1 (B) 111 NSCLC patients, validation set 2 and (C) 54 patients with NSCLC, validation set 3. The cut off for distinction between low and “normal” expression was based on the 33-percentile of expression values.

To further investigate the association between low HSP90 expression and NSCLC patient prognosis, we used three independent validation sets of NSCLC patients. In all three patient sets, the “low-risk”/“high-risk” patient distribution across patient cohorts was approximately two-thirds vs. one-third. Therefore, we used the 33-percentile of HSP90 expression as cut off for separation of patients with “normal” (i.e. high-risk) and “low” (i.e. low-risk) expression. For all three validation sets, low expression of HSP90 was correlated with improved overall survival. This correlation was significant for the first and third validation sets (P = 0.003 and P = 0.04), and borderline significant for the second set (P = 0.07) (Figure 3). Multivariate analysis revealed that HSP90 prognostic value was independent from the stage, histologic subtype, age and gender of patients (Table 3).

**Table 3:** Multivariate cox analysis of HSP90 expression and survival

	Validation set 1			Validation set 2			Validation set 3		
	HR	95% CI	P value	HR	95% CI	P value	HR	95% CI	P value
HSP90	0.41	0.22–0.75	0.004	0.52	0.27–0.96	0.036	0.24	0.05–1.07	0.061
STAGE	1.47	1.11–1.95	0.008	n/a			0.51	0.13–1.94	0.320
HISTOLOGY	0.84	0.59–1.19	0.320	0.71	0.41–1.21	0.210	0.48	0.18–1.29	0.140
AGE	0.99	0.95–1.03	0.550	n/a			1.00	0.94–1.07	0.900
GENDER	0.26	0.09–0.72	0.010	n/a			1.23	0.45–3.34	0.690

HR = Hazard Ratio; n/a = data not publicly available or incomplete

### Validation of Hsp90 as a viable molecular target in a panel of NSCLC cell lines

**Figure 4:**
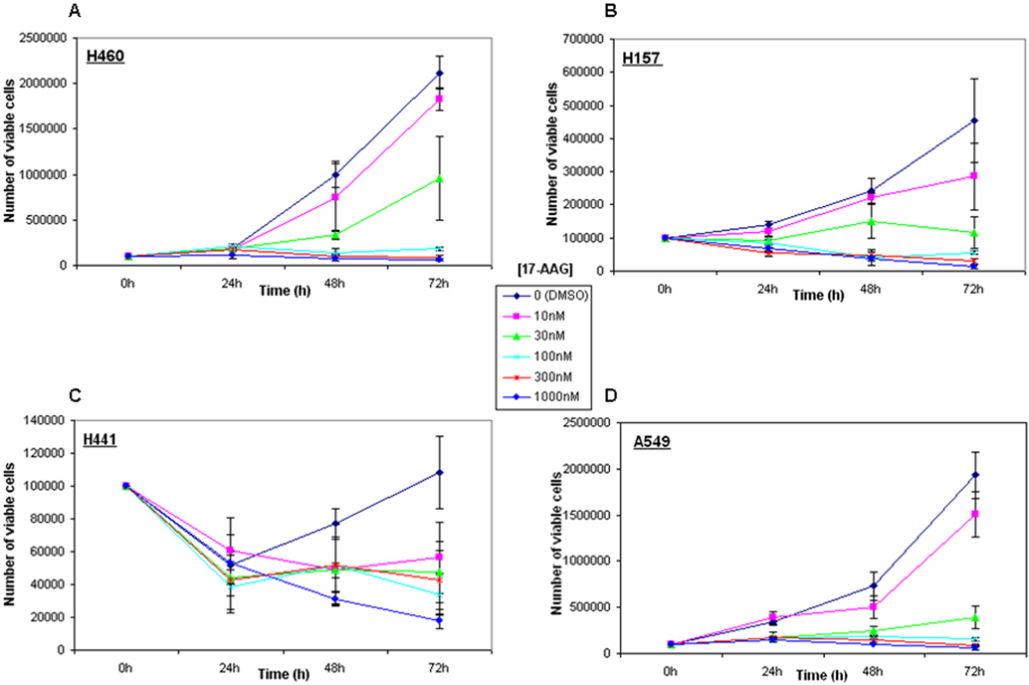
Sensitivity of a panel of wild type EGFR-expressing NSCLC cell lines to the Hsp90 inhibitor 17-AAG. (A–D) time- and dose-dependent inhibition of the in vitro growth of H460, H157, H441, and A549 NSCLC cell lines following exposure to 17-AAG. Cells were seeded at 105/well, and viable cell number was determined on subsequent days as described in Methods. 17-AAG concentrations at (H441) or above (A549, H460 & H157) 30 nM uniformly resulted in time-dependent loss of cell viability. The IC50 value of 17-AAG (continuous exposure for 72 h) for each cell line is as follows: H460 = 30 nM, H157 = 15 nM, H441 = 8 nM, and A549 = 20 nM.

Hsp90 is a molecular chaperone that stabilizes several oncoproteins, including EGFR, and constitutes a novel potential target for anticancer therapy. The data above showed that Hsp90 expression level is a prognostic indicator of long-term survival in a large series of NSCLC patients, and suggest that Hsp90 inhibitors may have broader utility in this disease than previously recognized. To examine this possibility, we tested whether pharmacologic inhibition of Hsp90 function would impact the *in vitro* cell growth of a panel of NSCLC cell lines. The growth of these cell lines, all bearing wild-type EGFR, was profoundly inhibited, in a dose- and time-dependent manner, by sub-micromolar concentrations of 17-AAG, an Hsp90 inhibitor [14] currently in phase II clinical trial (Figure 4). At 72 hours, the IC50 was below 50 nM 17-AAG in all cases, and higher drug concentrations consistently resulted in marked cytotoxicity.

## Discussion

Based on array-CGH data in NSCLC, it has been shown that multiple molecular carcinogenesis pathways exist that are most likely related to gender and smoking habits [15]. Furthermore, it was shown that there is a large overlap between aberrations observed in the adenocarcinoma and squamous cell carcinoma subtype, except for 3q gains which seem to be more specific for the squamous cell carcinoma subtype [16]. Various gene expression signatures have been correlated to survival of NSCLC patients [17]–[19]. In addition, molecular studies have allowed the development of personalized treatment approaches in several tumor types [20]–[22]. However, it is likely that many cancer-related target genes have not been identified yet. In this regard, integrated genome wide screening of copy number changes and gene expression using microarrays has been recently carried out in various tumor types to identify genes whose expression is affected by gene dosage [4], [23]–[26]. These studies aim to identify novel cancer-related genes and to define novel biomarkers for response or prognostic signatures. In both NSCLC and ductal pancreatic cancer, two focal amplifications of 8p12 and 20q11 have been studied in detail leading to two candidate genes (WHSC1L1 and TPX2) important in these diseases [16]

We here performed an integrated genome wide screening of gene copy number changes and gene expression in 32 radically resected NSCLC patients, in order to identify novel NSCLC-related genes. By using ACE-it, a novel informatics tool for integration of gene dosage and gene expression data, we identified 359 transcripts to be significantly affected by copy number. A cox survival analysis on all 359 genes revealed no significant relation after multiple testing. The top list of genes related to survival mainly included genes residing on gained regions 3q and 5p. These regions cover many genes and to pinpoint the gene of most importance is a challenging task. Further investigations should elucidate the importance of these genes in relation to NSCLC, in particular of those in the top list of correlation with survival such as SLC45A2, WDR70 and NIPBL (see supplementary table S1). In this paper we focused on the recurrent deletion on chromosome 14 and the gene HSP90, which was also in the top list of relation with survival and not previously investigated in detail. Deletion of region 14q32.2-33 was correlated with improved survival, further suggesting that it may contain one or more genes related to NSCLC progression. Deletion of this region has been previously described by one group reporting genomic aberrations in NSCLC, but was not investigated in further detail [27]. Out of the 109 genes mapping to the 14q32.2-33 region, HSP90 was the only gene with significantly lower expression in patients harboring the 14q32.2-33 deletion. In the initial series of 29 patients (3 patients excluded from survival analysis), we observed improved survival in patients with lower levels of HSP90. The association between HSP90 expression levels and NSCLC patient prognosis was confirmed to be significant in three independent validation sets of NSCLC patients. Multivariate analysis including stage, histology, age and gender showed that HSP90 remained independently related to survival.

A critical issue in defining “low” and “normal” expression is the choice of an appropriate cut off value. In the initial analyses we used the median of expression ratios as cut off between “low” and “normal” expression, since the low-risk and high-risk separation of patients was equal. However, in the validation sets the low-risk and high-risk survival groups were not equally balanced (two thirds versus one third). Consequently the cut off values used in these data sets was not the median value, but the 33-percentile.

In this study, using a genome wide integrative analysis of gene copy number and expression we were able to identify expression of HSP90 as an important gene in early stage NSCLC patients. HSP90 is a chaperone protein involved in the stabilization of multiple oncoproteins such as EGFR, Her-2 and Akt [28]. Recent work has shown that HSP90 plays a role in maintaining the active conformation of EGFR and in particular EGFR mutants [29], [30]. We show here that several NSCLC cell lines bearing wild-type EGFR are sensitive to HSP90 inhibition, indicating that the inhibitory effect of 17-AAG can not be solely attributed to mutant EGFR. HSP90 has been recently recognized as a potential cancer therapeutic target and investigations of HSP90 inhibitors are ongoing [31], [32]. In this regard, glioblastoma cells overexpressing EGFR, but resistant to inhibition by EGFR kinase inhibitors, were sensitive to HSP90 inhibition [33]. Therefore, while Hsp90 may be required to stabilize over-expressed or mutated EGFR, our data support a more wide-ranging and complex role for Hsp90 in mediating NSCLC growth and survival. The low nanomolar sensitivity observed in the 4 cell lines tested in our experiments, is in agreement with other published reports of highly 17-AAG sensitive tumor cell lines [34]–[36]. These concentrations are readily achievable in patients for prolonged time periods using current scheduling and dosing regimens [37].

In summary, the observation that HSP90 expression level is a prognostic factor for NSCLC patient survival (independent of EGFR mutational status), coupled with the extreme sensitivity of EGFR wild type NSCLC cells to the Hsp90 inhibitor 17-AAG, suggests that Hsp90 inhibitors may have greater clinical utility in NSCLC than has been previously considered and warrants further investigation of the dependence of other proto-oncogenes on this chaperone protein in NSCLC.

## Supporting Information

Table S1 (0.03 MB PDF)
