# Supplementary material for: Integration of Gene Dosage and Gene Expression in Non-Small Cell Lung Cancer, Identification of HSP90 as Potential Target
Source: PLoS One. 2008 Mar 5;3(3):e1722. doi: 10.1371/journal.pone.0001722 (PMC2254495; doi:10.1371/journal.pone.0001722)
Supplement: Table S1 — (0.03 MB PDF) [file pone.0001722.s001.pdf]

Supplementary Table S1: Genes identified by ACE-it (359) ordered according to their relation with survival (Univariate cox regression analysis)

							ACE-it		Univariate Cox regression	
ID	Gene	Chromosome	Start	End	Clone	Gene name	Raw p-value	Adj p-value	Raw p-value	Adj p-value
8984	BC032907	5	10405817	10406728	BC032907		0.001207	0.021651	0.003407233	0.80291037
9076	NM_016180	5	33980476	34020607	NM_016180	SLC45A2	0.000581	0.014974	0.005470466	0.80291037
9118	BC032711	5	36912618	37100061	BC009608	NIPBL	0.001968	0.030332	0.010061377	0.80291037
9178	U09410	5	43157626	43212050	NM_018038		0.004104	0.045148	0.017059899	0.80291037
9117	NM_015384	5	36912618	37100061	BC009608	NIPBL	0.004916	0.049385	0.020917942	0.80291037
9156	I_958346	5	40861121	40865014	NM_000997	RPL37	0.001207	0.021651	0.024471588	0.80291037
7121	I_928694	3	161699072	161701137	AK055675		0.000277	0.010568	0.026681546	0.80291037
9125	NM_018034	5	37415071	37788535	AK022353	WDR70	0.004916	0.049385	0.028352189	0.80291037
7442	NM_153690	3	195889757	195889816	NM_153690	FAM43A	0.002964	0.037767	0.03058387	0.80291037
9001	BC035585	5	14323828	14561785	AK023551		0.004104	0.045148	0.034076938	0.80291037
8956	THC1570157	5	5491079	5491578	BC004902		0.000986	0.018905	0.034085862	0.80291037
6908	NM_006153	3	138063770	138150670	AK021677	NCK1	0.000655	0.015855	0.037417616	0.80291037
7103	NM_024996	3	159844914	159893141	NM_020169	GFM1	0.002386	0.033738	0.042916755	0.80291037
9065	NM_006713	5	32621429	32637821	NM_006713	SUB1	0.004633	0.047867	0.043110532	0.80291037
6979	NM_004267	3	144320871	144324927	AK097380	CHST2	0.000178	0.008665	0.045467411	0.80291037
9158	NM_000997	5	40866375	40870455	NM_000997	RPL37	0.003941	0.043729	0.048220053	0.80291037
9090	AK001962	5	34951572	34961542	NM_133377	BXDC2	0.001114	0.020501	0.048424286	0.80291037
22961	NM_005348	14	101617145	101623135	NM_005348	HSP90AA1	0.004917	0.049385	0.049117965	0.80291037
7122	NM_002268	3	161700644	161766078	AY077740	KPNA4	6.20E-05	0.005471	0.052006702	0.80291037
7073	NM_020865	3	155476151	155524988	NM_020865	DHX36	0.000115	0.007278	0.05558956	0.80291037
9089	NM_018321	5	34951227	34961857	NM_133377	BXDC2	0.001264	0.022344	0.057362309	0.80291037
6866	NM_032169	3	133778495	133778554	NM_032169	ACAD11	6.60E-05	0.005471	0.057585245	0.80291037
7214	AK023267	3	173951210	174021974	AF085992	ECT2	0.000125	0.00775	0.070261095	0.80291037
6884	NM_021203	3	135007306	135022314	NM_016577	SRPRB	8.20E-05	0.005789	0.070669664	0.80291037
6956	NM_001679	3	143078168	143128080	NM_001679	ATP1B3	3.00E-06	0.00208	0.077140471	0.80291037
6957	BU625774	3	143078168	143128080	NM_001679	ATP1B3	3.00E-06	0.00208	0.079933706	0.80291037
7352	I_945862	3	187784398	187786297	NM_016306	DNAJB11	0.003702	0.042383	0.090016222	0.80291037
25460	NM_022764	16	85121950	85146325	NM_022764	FLJ12998	0.000664	0.015855	0.092026559	0.80291037
6940	BF746243	3	141139228	141139627	AF052176		0.002424	0.033746	0.095895347	0.80291037
7318	NM_004443	3	185762251	185782907	NM_004443	EPHB3	0.000581	0.014974	0.097690333	0.80291037
7467	NM_005017	3	197453508	197502905	NM_005017	PCYT1A	0.000752	0.016228	0.100157297	0.80291037
9025	NM_033414	5	16504621	16518910	NM_033414	ZNF622	0.000917	0.018362	0.102420132	0.80291037
7170	NM_024947	3	171297360	171382218	AK022455	PHC3	0.000324	0.010812	0.102767371	0.80291037
7123	U93240	3	161700644	161766078	AY077740	KPNA4	0.000155	0.008471	0.105353549	0.80291037
28461	I_962019	19	12671002	12694104	NM_013433	TNPO2	0.000838	0.017357	0.105369	0.80291037
7065	AK026659	3	154039777	154041927	AK026659		0.000942	0.018362	0.110855481	0.80291037
28381	NM_023008	19	10524651	10537702	NM_023008	FLJ12949	0.001852	0.029163	0.114477944	0.80291037
7420	BC043443	3	194793709	194895796	NM_130837	OPA1	0.000387	0.011731	0.114638288	0.80291037
9063	AK025481	5	32390206	32439135	NM_016107	ZFR	0.000445	0.012346	0.116415456	0.80291037
7449	I_3558062	3	196731799	196751283	NM_006241	PPP1R2	0.000254	0.010153	0.12107484	0.80291037
7146	NM_007217	3	168884398	168935383	NM_007217	PDCD10	0.000724	0.01603	0.123949276	0.80291037
7019	NM_016094	3	150942041	150942100	NM_016094	COMMD2	0.000372	0.01158	0.124959368	0.80291037
6852	NM_014382	3	132095730	132204787	NM_014382	ATP2C1	1.30E-05	0.003026	0.127763688	0.80291037
28382	NM_001800	19	10538138	10540631	NM_001800	CDKN2D	0.003052	0.038079	0.128668221	0.80291037
28347	I_1850394	19	9580140	9592916	NM_152289	ZNF561	0.000245	0.010153	0.133348831	0.80291037
7441	NM_018385	3	195842811	195874245	NM_018385	LSG1	0.004367	0.046409	0.134471137	0.80291037
28387	AF141870	19	10642171	10661429	NM_012218	ILF3	0.003052	0.038079	0.134577185	0.80291037
7115	NM_005496	3	161634283	161634342	NM_005496	SMC4L1	0.000181	0.008665	0.137597602	0.80291037
7064	NM_002563	3	154035420	154038539	NM_002563	P2RY1	0.000277	0.010568	0.138154561	0.80291037
7124	A_32_BS214969	3	161700644	161766078	AY077740		0.000422	0.01229	0.145734415	0.80291037
28380	NM_032885	19	10515566	10525102	NM_032885	ATG4D	0.001098	0.020501	0.153472002	0.80291037
28344	NM_006631	19	9384761	9384820	NM_006631	ZNF266	0.001565	0.02588	0.161358859	0.80291037
6864	NM_001099	3	133518976	133560324	NM_001099	ACPP	0.000752	0.016228	0.162812395	0.80291037
7472	AK097068	3	197683975	197686377	AK097068		0.000655	0.015855	0.162949619	0.80291037

28396	ENST00000324954	19	10990557	11030769	NM_003072	SMARCA4	0.00437	0.046409	0.164191209	0.80291037
7393	NM_003722	3	190938080	191095193	NM_003722	TP73L	4.90E-05	0.005042	0.165245007	0.80291037
7478	AK000529	3	197927514	197943538	NM_017861	PIGX	0.000322	0.010812	0.166186283	0.80291037
7398	AF115546	3	191506765	191522966	NM_021101	CLDN1	0.00162	0.026081	0.170783	0.80291037
6854	AK074692	3	132096132	132218260	NM_014382	ATP2C1	2.20E-05	0.003587	0.176028836	0.80291037
7213	NM_018098	3	173951210	174021974	AF085992	ECT2	0.001426	0.023796	0.177058704	0.80291037
7286	NM_024871	3	185016364	185026120	NM_024871	MAP6D1	0.000724	0.01603	0.180939946	0.80291037
6977	BC006474	3	144203171	144258427	BC040051		7.00E-05	0.005471	0.186619356	0.80291037
7395	I_3559341	3	191095561	191097771	NM_003722	TP73L	1.60E-05	0.003026	0.189199733	0.80291037
7282	THC1431796	3	184959382	184960057	NM_018023	YEATS2	0.000938	0.018362	0.194011508	0.80291037
16298	NM_016307	9	129507473	129564512	NM_016307	PRRX2	0.003873	0.043729	0.195969401	0.80291037
7342	U87836	3	187132250	187138508	NM_004593	SFRS10	0.001432	0.023796	0.198123978	0.80291037
7038	Y15268	3	151941598	151963862	NM_152394	SIAH2	1.70E-05	0.003078	0.201102262	0.80291037
7479	NM_017861	3	197927549	197950151	NM_017861	PIGX	0.000861	0.017499	0.202958467	0.80291037
30701	NM_017896	20	61047021	61047080	NM_017896	C20orf11	0.001311	0.022947	0.20594529	0.80291037
7477	NM_032898	3	197922231	197927474	NM_032898	C3orf34	0.003094	0.038079	0.20961313	0.80291037
28395	NM_003072	19	10932606	11033953	NM_003072	SMARCA4	0.001629	0.026102	0.210021887	0.80291037
7116	I_1961525	3	161635775	161637287	BU172301		0.000725	0.01603	0.210237216	0.80291037
9109	NM_032637	5	36187940	36219902	NM_005983	SKP2	0.004585	0.047788	0.210430359	0.80291037
28198	NM_018708	19	4742719	4746582	AI826903	FEM1A	0.003143	0.038293	0.212896829	0.80291037
7081	NM_003875	3	157071022	157138220	NM_003875	GMPS	0.000661	0.015855	0.213124018	0.80291037
6942	BX097190	3	141778218	141778943	AK056386		0.003488	0.040969	0.214991121	0.80291037
7445	THC1598616	3	196270302	196473373	NM_152531	C3orf21	0.004367	0.046409	0.215237502	0.80291037
6945	NM_018155	3	142143347	142178943	NM_152616	SLC25A36	1.30E-05	0.003026	0.217420265	0.80291037
7222	NM_030921	3	178222171	178222230	NM_030921		0.000697	0.01603	0.222723493	0.80291037
24324	NM_152891	16	2773954	2776625	NM_152891	PRSS33	0.000636	0.015855	0.224394375	0.80291037
28167	NM_030662	19	4041294	4075126	NM_020224	MAP2K2	0.000426	0.01229	0.228565475	0.80291037
30670	THC1581610	20	60145337	60147890	NM_152255		0.00162	0.026081	0.233552977	0.80291037
6853	BC028139	3	132095868	132218251	NM_014382	ATP2C1	2.20E-05	0.003587	0.233589192	0.80291037
28126	NM_020170	19	3136598	3160615	NM_020170	NCLN	0.000487	0.013311	0.237369064	0.80291037
6954	BC008627	3	142939743	142947940	NM_014245	RNF7	3.00E-06	0.00208	0.237398136	0.80291037
7341	NM_004593	3	187117238	187138479	NM_004593	SFRS10	0.003702	0.042383	0.239570663	0.80291037
7045	THC1444068	3	152470108	152470607	AF318318		0.000115	0.007278	0.239853139	0.80291037
7471	AB018337	3	197570058	197647610	AK092964	UBXD7	0.003094	0.038079	0.241497419	0.80291037
6549	AK093100	3	102929004	102966576	AK022267	PDCL3	3.70E-05	0.004288	0.242166262	0.80291037
9188	NM_012343	5	43638573	43741468	AK096668	NNT	0.004144	0.045148	0.242801462	0.80291037
7148	NM_014498	3	169209917	169296446	AK000777	GOLPH4	0.003522	0.040969	0.244263082	0.80291037
28450	NM_020714	19	12552139	12582606	NM_153358	ZNF490	0.000445	0.012346	0.25110729	0.80291037
28091	BC018621	19	2272513	2279600	NM_016199	LSM7	0.003941	0.043729	0.251356353	0.80291037
7473	NM_152617	3	197686939	197718949	BE740187	RNF168	0.00274	0.036066	0.252908533	0.80291037
7292	BC015334	3	185200873	185218429	NM_005688		0.000724	0.01603	0.255837663	0.80291037
7455	NM_005781	3	197078944	197079003	NM_005781	TNK2	0.000187	0.008665	0.258170157	0.80291037
28469	NM_005809	19	12768603	12773694	NM_002229	PRDX2	2.40E-05	0.003622	0.259516812	0.80291037
28399	BC014514	19	11061132	11105490	NM_000527	LDLR	0.002103	0.031336	0.262018261	0.80291037
9088	NM_133377	5	34944049	34951540	NM_133377	RAD1	0.001264	0.022344	0.262409796	0.80291037
7363	NM_002916	3	187990386	188006992	NM_002916	RFC4	0.002619	0.034848	0.263299773	0.80291037
7470	THC1515738	3	197562843	197563799	AB018337	UBXD7	0.002424	0.033746	0.264202561	0.80291037
28596	THC1494039	19	16602578	16631941	NM_024104	MGC2747	4.40E-05	0.004773	0.26751167	0.80291037
28343	AB095928	19	9383638	9406980	NM_006631	ZNF266	0.002158	0.031376	0.267710235	0.80291037
28337	NM_020933	19	9112056	9135138	NM_020933	ZNF317	3.50E-05	0.004288	0.268683778	0.80291037
28353	NM_006221	19	9806880	9821368	NM_006221	PIN1	3.40E-05	0.004288	0.269750844	0.80291037
7394	AF061512	3	190989857	191074066	NM_003722	TP73L	7.90E-05	0.00577	0.272812252	0.80291037
28470	NM_181738	19	12768637	12773694	NM_002229	PRDX2	7.70E-05	0.00577	0.273609798	0.80291037
7335	AK074357	3	186786713	186831594	NM_021627	SENP2	0.004144	0.045148	0.274489857	0.80291037
7447	NM_012287	3	196480321	196645069	NM_012287	CENTB2	0.000935	0.018362	0.275539087	0.80291037
7224	NM_024665	3	178223690	178397751	AF268194	TBL1XR1	0.001612	0.026081	0.27733544	0.80291037
7174	NM_002740	3	171422920	171503920	NM_005414	PRKCI	0.002494	0.033808	0.27888811	0.80291037
7411	NM_178496	3	193997304	194000684	BC036194	C3orf59	0.000221	0.009371	0.279342401	0.80291037
7260	NM_003106	3	182912404	182914923	BC013923	SOX2	0.002943	0.037635	0.279510235	0.80291037
6880	AK026229	3	134802100	134863436	NM_007027	TOPBP1	5.00E-06	0.002995	0.282060376	0.80291037
28245	NM_032306	19	6323444	6326077	NM_032306	ALKBH7	0.000938	0.018362	0.28643524	0.80291037

7034	BC039424	3	151747246	151803713	AI560242		2.40E-05	0.003622	0.287344892	0.80291037
6550	AK054664	3	102980750	103025205	NM_145037	FAM55C	0.000614	0.015566	0.28945197	0.80291037
28231	THC1555999	19	5855869	5865711	NM_004058	CAPS	0.001367	0.023135	0.290810563	0.80291037
6855	AK027806	3	132215419	132228356	NM_014065	ASTE1	0.003927	0.043729	0.291143652	0.80291037
30699	A_32_BS19917	20	61006886	61016130	NM_022105	DIDO1	0.00162	0.026081	0.291704381	0.80291037
28212	BC029496	19	5224073	5237404	NM_002850	PTPRS	0.001064	0.020096	0.293628379	0.80291037
9110	NM_153013	5	36228745	36278015	BC033402	FLJ30596	0.001612	0.026081	0.294554583	0.80291037
7067	THC1417289	3	154363631	154364489	BC034800		9.90E-05	0.006728	0.294620174	0.80291037
28504	NM_017721	19	13864993	13902699	NM_017721	CC2D1A	0.002003	0.030734	0.29959153	0.80291037
7021	BC017878	3	151013150	151162653	NM_007282	RNF13	4.50E-05	0.00482	0.299841262	0.80291037
7485	I_1100153	3	198144367	198147893	NM_152699	SENP5	0.002424	0.033746	0.303582443	0.80291037
6930	NM_023067	3	140146123	140146182	NM_023067	FOXL2	0.000433	0.01229	0.305101336	0.80291037
7227	AF279780	3	179725959	179727750	AF279780		0.001114	0.020501	0.305892782	0.80291037
28553	NM_014371	19	15368989	15369048	NM_014371	AKAP8L	0.001008	0.01923	0.308605586	0.80291037
7022	NM_007282	3	151013255	151162621	NM_007282	RNF13	1.40E-05	0.003026	0.309568822	0.80291037
7421	NM_130837	3	194793869	194892618	NM_130837	OPA1	0.002327	0.033302	0.30979187	0.80291037
28477	NM_004461	19	12894293	12905522	NM_004461	FARSLA	0.000359	0.011452	0.310588755	0.80291037
28616	NM_024050	19	17281322	17292169	NM_024050	DDA1	0.004367	0.046409	0.311767892	0.80291037
7156	NM_018657	3	170973900	170988034	NM_032487	MYNN	1.60E-05	0.003026	0.312115937	0.80291037
7242	NM_021629	3	180599704	180652073	NM_021629	GNB4	0.003702	0.042383	0.315284809	0.80291037
7197	AK026225	3	172988765	173010938	AF318333		0.000295	0.010736	0.318086119	0.80291037
6946	AL136803	3	142143347	142178943	NM_152616	SLC25A36	0.003488	0.040969	0.32032502	0.80291037
6959	NM_006286	3	143153036	143230197	NM_006286	TFDP2	0.000864	0.017499	0.321396899	0.80291037
28361	NM_020230	19	10082904	10082963	NM_020230	PPAN	0.00383	0.043572	0.32152213	0.80291037
28705	NM_017660	19	19357832	19479060	NM_017660	GATAD2A	0.004367	0.046409	0.324490552	0.80291037
7290	I_1152539	3	185120428	185183087	NM_005688	ABCC5	0.000487	0.013311	0.325028234	0.80291037
7240	AK091332	3	180596579	180598815	NM_021629		0.004633	0.047867	0.325216761	0.80291037
7281	NM_018023	3	184898308	185013114	NM_018023	YEATS2	5.20E-05	0.005042	0.330560244	0.80291037
7033	NM_032025	3	151747213	151784727	AI560242	EIF2A	0.001772	0.028154	0.330738388	0.80291037
7066	NM_002886	3	154362708	154364173	BC034800	RAP2B	0.002547	0.034405	0.331573522	0.80291037
6878	NM_017548	3	134775183	134791810	NM_017548	CDV3	0.000495	0.013433	0.335910814	0.80291037
6937	NM_178177	3	140761717	140879538	NM_025093	NMNAT3	0.000178	0.008665	0.337465211	0.80291037
28394	NM_138358	19	10900397	10905102	NM_138358	LOC90580	0.000146	0.008207	0.338510447	0.80291037
7068	I_928480	3	154364171	154367654	BC034800		0.000422	0.01229	0.338660708	0.80291037
7308	NM_002808	3	185500798	185500857	NM_002808	PSMD2	0.000214	0.009371	0.340865729	0.80291037
7334	NM_021627	3	186786713	186831594	NM_021627	SENP2	0.000757	0.016253	0.344400542	0.80291037
7184	AL110170	3	172066750	172070772	AL110170	RPL22L1	5.90E-05	0.005471	0.34567335	0.80291037
7490	NM_138487	3	198157804	198159194	NM_138487		0.00274	0.036066	0.347508344	0.80291037
6966	NM_001184	3	143650449	143780349	AK022009	ATR	0.000131	0.008008	0.349352121	0.80291037
7444	AK075551	3	196270302	196473373	NM_152531	C3orf21	0.001227	0.021911	0.350127798	0.80291037
7036	NM_016275	3	151803764	151828452	NM_016275	SELT	0.000725	0.01603	0.351595564	0.80291037
25448	BC039013	16	84390692	84398144	NM_001861	COX4I1	0.002065	0.031292	0.352979223	0.80291037
28334	NM_144693	19	8781372	8794565	NM_144693	ZNF558	0.002484	0.033808	0.353944737	0.80291037
6934	NM_004766	3	140559125	140591159	NM_004766	COPB2	0.000751	0.016228	0.362183114	0.80291037
7397	NM_021101	3	191506765	191522966	NM_021101	CLDN1	0.004633	0.047867	0.362355854	0.80291037
28463	NM_024038	19	12702444	12706589	NM_024038	MGC2803	0.00031	0.010812	0.365242585	0.80291037
28530	NM_138501	19	14535850	14535909	NM_138501	GPSN2	0.000209	0.009371	0.367266904	0.80291037
14997	NM_016623	8	130922890	131021182	BG215708	FAM49B	0.000532	0.014223	0.3688312	0.80291037
6900	NM_018133	3	137351174	137351233	NM_018133	MSL2L1	2.90E-05	0.003858	0.368990292	0.80291037
28471	NM_006397	19	12779241	12779300	NM_006397	RNASEH2A	0.001046	0.019847	0.369025658	0.80291037
7261	BC013923	3	182912404	182914923	BC013923		0.002327	0.033302	0.372353208	0.80526989
28378	NM_012289	19	10457765	10474481	NM_012289	KEAP1	0.002699	0.035786	0.375950561	0.806733696
28349	NM_017656	19	9624293	9646736	NM_017656	ZNF562	0.000725	0.01603	0.377524404	0.806733696
7289	NM_005688	3	185120419	185218411	NM_005688	ABCC5	0.000372	0.01158	0.385454187	0.813074702
28348	NM_152289	19	9580140	9592916	NM_152289	ZNF561	2.60E-05	0.003622	0.386534628	0.813074702
6879	BC007338	3	134775518	134788884	NM_017548	CDV3	7.00E-05	0.005471	0.389491606	0.813074702
28308	AK096659	19	7929458	7933376	NM_006351	ELAVL1	1.30E-05	0.003026	0.389551111	0.813074702
28114	NM_173480	19	2851896	2869467	BC028974	LOC126295	0.001207	0.021651	0.396826481	0.823472293
6886	AK055102	3	135025774	135030312	NM_016577	RAB6B	0.001283	0.022574	0.409025385	0.837824509
28430	NM_152357	19	11786099	11807424	AK021474	ZNF440	0.002135	0.031376	0.411520943	0.837824509
28153	NM_172251	19	3713671	3718568	NM_172251	MRPL54	0.003143	0.038293	0.418058309	0.837824509

28042	NM_152482	19	1424203	1430158	NM_152482	C19orf25	0.001207	0.021651	0.423264253	0.837824509
6980	I_1971042	3	144466754	144895544	BC035779		0.000278	0.010568	0.425397389	0.837824509
30681	BC018140	20	60395553	60396978	AK057740	RPS21	0.000337	0.010953	0.426211832	0.837824509
28010	NM_002085	19	1054963	1057793	NM_002085	GPX4	0.003941	0.043729	0.429684146	0.837824509
7350	NM_018138	3	187746832	187767831	NM_018138	TBCCD1	0.000862	0.017499	0.433855277	0.837824509
7231	NM_006218	3	180399304	180435197	NM_006218	PIK3CA	2.60E-05	0.003622	0.434319577	0.837824509
31094	I_3590203	21	44387576	44390032	NM_004649	C21orf33	0.003927	0.043729	0.436324811	0.837824509
7456	ENST00000316664	3	197083404	197107763	NM_005781	TNK2	0.000613	0.015566	0.436832551	0.837824509
7475	NM_182627	3	197769335	197783771	NM_032891	WDR53	0.003488	0.040969	0.438271024	0.837824509
6865	AB014578	3	133665241	133740583	AK023168		9.00E-06	0.002995	0.440200702	0.837824509
28036	NM_001018	19	1389379	1391492	NM_170711	RPS15	0.000724	0.01603	0.441683951	0.837824509
28398	NM_000527	19	11061132	11105490	NM_000527	LDLR	0.00437	0.046409	0.442268318	0.837824509
28137	THC1562847	19	3474304	3474821	BG029787		0.002494	0.033808	0.445570764	0.837824509
28111	NM_152303	19	2770891	2786469	NM_152303	ZNF554	0.002802	0.036211	0.4457087	0.837824509
6952	NM_006506	3	142688624	142816902	NM_006506	RASA2	9.60E-05	0.006651	0.445750644	0.837824509
6947	AL049246	3	142171517	142181475	NM_152616	SLC25A36	1.30E-05	0.003026	0.450746571	0.842802183
7438	NM_138399	3	195812948	195813007	NM_138399	TMEM44	0.00078	0.016647	0.454639436	0.845651388
6891	BC021700	3	135359145	135424067	AL133627		9.00E-06	0.002995	0.458798466	0.845651388
6905	NM_005862	3	137537674	137832838	AF251189	STAG1	3.00E-06	0.00208	0.466670564	0.845651388
28181	NM_003025	19	4311358	4351574	NM_003025	SH3GL1	0.004404	0.046409	0.47025983	0.845651388
28423	NM_032377	19	11524858	11531032	BC039523	ELOF1	7.00E-06	0.002995	0.470702418	0.845651388
6890	AL133627	3	135354185	135355696	AL133627		0.000655	0.015855	0.471721628	0.845651388
28116	I_1854559	19	2884209	2895941	AL365370	ZNF77	0.002802	0.036211	0.47316269	0.845651388
28531	NM_004146	19	14537866	14543890	NM_138501	NDUFB7	0.000752	0.016228	0.477607013	0.845651388
24289	NM_004424	16	2213567	2225752	NM_004424	E4F1	0.004585	0.047788	0.479943904	0.845651388
28467	NM_013312	19	12733872	12747357	NM_013312	HOOK2	0.002103	0.031336	0.480180374	0.845651388
7403	AF029213	3	191714593	191849260	AF116662		0.000981	0.018905	0.481398489	0.845651388
28366	NM_015956	19	10223640	10231736	NM_146388	MRPL4	0.000107	0.007036	0.482619341	0.845651388
7256	AF075028	3	182181996	182183240	AF075028		0.004144	0.045148	0.484144927	0.845651388
7069	BQ717518	3	154370613	154371105	BC034800		0.000134	0.008032	0.485248429	0.845651388
7466	BC042043	3	197449550	197502932	NM_005017		0.00043	0.01229	0.491655548	0.84789921
7047	NM_023915	3	152494572	152517438	NM_023915	GPR87	0.000827	0.017357	0.49214486	0.84789921
6851	NM_014602	3	131880464	131948394	NM_014602	PIK3R4	7.00E-05	0.005471	0.493876082	0.84789921
6933	NM_020191	3	140545517	140558683	NM_020191	MRPS22	9.00E-06	0.002995	0.501081075	0.84789921
28307	NM_006351	19	7897590	7914656	BC022423	TIMM44	0.000293	0.010736	0.50396045	0.84789921
28222	NM_015414	19	5641367	5642707	NM_004793	RPL36	0.004404	0.046409	0.5049455	0.84789921
15015	D63477	8	132985535	133095071	AK002123	KIAA0143	0.001825	0.028867	0.50561491	0.84789921
6984	I_951890	3	145191189	145193901	BC037293		0.004414	0.046409	0.508237346	0.84789921
6898	NM_002718	3	137167221	137348090	NM_002718	PPP2R3A	5.00E-05	0.005042	0.508440573	0.84789921
6951	AK055693	3	142648014	142651323	NM_152535	ZBTB38	0.002424	0.033746	0.510209921	0.84789921
9062	AY028703	5	32265402	32348832	NM_019061	MTMR12	0.002053	0.03123	0.512518464	0.84789921
28480	NM_005053	19	12917654	12925455	NM_005053	RAD23A	0.000838	0.017357	0.515300332	0.848215256
6953	AK025305	3	142688624	142816902	NM_006506		0.000178	0.008665	0.51923974	0.848215256
7275	AK074235	3	184453717	184473889	AB020668	B3GNT5	0.000193	0.008802	0.52355269	0.848215256
28094	NM_012458	19	2376617	2378906	NM_012458	TIMM13	0.004916	0.049385	0.525492221	0.848215256
28498	NM_014047	19	13746249	13750289	NM_014047	HSPC023	0.002124	0.031376	0.526688783	0.848215256
7094	BX095281	3	158362289	158363051	AV696265		0.00482	0.049385	0.527681349	0.848215256
28037	I_3554426	19	1389379	1391492	NM_170711	DAZAP1	0.002802	0.036211	0.529248516	0.848215256
28537	ENST00000248058	19	14812760	14813689	NM_017506	OR7A10	0.003032	0.038079	0.539748831	0.858812663
28122	L40630	19	3070198	3072108	NM_152294	GNA11	0.004916	0.049385	0.540645298	0.858812663
16311	AK023462	9	129971472	129973770	NM_020960	GPR107	0.00236	0.033501	0.551347778	0.859169314
6868	NM_024818	3	133861786	133879473	NM_024818	UBE1DC1	1.30E-05	0.003026	0.553099953	0.859169314
7337	NM_006548	3	186844283	186899183	NM_006548	IGF2BP2	0.00162	0.026081	0.553893709	0.859169314
28612	NM_014173	19	17239286	17251144	NM_152363	HSPC142	0.00236	0.033501	0.554487198	0.859169314
28440	NM_003437	19	12160408	12160467	NM_003437	ZNF136	0.001701	0.027142	0.555506431	0.859169314
28429	BC016371	19	11786099	11807424	AK021474	ZNF440	0.00142	0.023796	0.557018799	0.859169314
30682	NM_001024	20	60395555	60396970	AK057740	RPS21	0.000255	0.010153	0.559774305	0.859169314
28607	AK074683	19	17187179	17190635	NM_018467	MDS032	0.000398	0.01195	0.560015653	0.859169314
28016	NM_001687	19	1192734	1195904	NM_001687	ATP5D	0.001968	0.030332	0.567945722	0.859654139
30668	AF165185	20	60143131	60143547	NM_144703		0.002619	0.034848	0.571936002	0.859654139
28287	I_2031437	19	7600678	7602641	BC038715	PCP2	0.000184	0.008665	0.575314302	0.859654139

28352	NM_024292	19	9799592	9801791	NM_024292	UBL5	0.000382	0.011731	0.575841462	0.859654139
28210	AK093006	19	5064893	5067259	AL831872		0.004404	0.046409	0.577800143	0.859654139
28433	AL136732	19	11896885	11922588	AL136732	ZNF700	0.002941	0.037635	0.578354181	0.859654139
7234	BC020500	3	180524253	180536029	NM_016331	ZNF639	0.000581	0.014974	0.579387922	0.859654139
7223	A_32_BS2342	3	178223690	178397751	AF268194		0.002311	0.033302	0.579488305	0.859654139
6887	NM_016577	3	135029996	135097375	NM_016577	RAB6B	0.002141	0.031376	0.585062322	0.864351332
9185	AK025310	5	43481203	43489940	NM_022483	FLJ21657	0.003941	0.043729	0.589782527	0.8677538
28350	THC1515004	19	9630974	9631611	NM_017656	ZNF562	0.000107	0.007036	0.597635191	0.870348969
7297	BC013590	3	185344652	185344711	BC013590		0.003321	0.039917	0.598624901	0.870348969
24308	THC1512019	16	2527853	2556608	NM_002613	PDPK1	0.000724	0.01603	0.602459337	0.870348969
7271	THC1573058	3	184229392	184230801	NM_020166	MCCC1	0.000445	0.012346	0.605772577	0.870348969
6919	NM_015396	3	139497519	139497578	NM_015396	ARMC8	0.001203	0.021651	0.607823778	0.870348969
28456	NM_013407	19	12638667	12654034	NM_016145	DHPS	0.002103	0.031336	0.609540801	0.870348969
24339	AK091002	16	3002458	3004507	NM_020982	CLDN9	0.001367	0.023135	0.612140969	0.870348969
7338	AW966840	3	186844283	186850013	AK001051		0.000337	0.010953	0.612358229	0.870348969
24854	NM_014699	16	30993258	31002335	NM_014699	ZNF646	0.00482	0.049385	0.613757226	0.870348969
6869	NM_153240	3	133882151	133923974	NM_152530	NPHP3	3.60E-05	0.004288	0.615791057	0.870348969
7268	AF456425	3	184144618	184181055	BC013163	DCUN1D1	4.30E-05	0.004773	0.618214449	0.870348969
28453	THC1509243	19	12604352	12605659	BU184121		0.000166	0.008545	0.63016651	0.881830409
7402	NM_002182	3	191714593	191849260	AF116662	IL1RAP	0.003702	0.042383	0.633257347	0.881830409
6981	NM_173653	3	144466754	144895544	BC035779	SLC9A9	0.000373	0.01158	0.633738846	0.881830409
28486	NM_017722	19	13076710	13088437	NM_005583	TRMT1	0.003499	0.040969	0.640788295	0.887028911
6860	NM_007208	3	132663726	132704527	NM_007208	MRPL3	0.00043	0.01229	0.644832401	0.887028911
7039	THC1434456	3	152071456	152072040	NM_052995	USH3A	0.000942	0.018362	0.644887314	0.887028911
6926	BC016050	3	139738460	139795880	NM_024491	CEP70	0.000153	0.008471	0.650408964	0.891209229
28581	NM_014077	19	16157223	16163855	NM_014077	FAM32A	0.00343	0.04095	0.654322868	0.892056601
28005	NM_033420	19	962132	972160	NM_033420	C19orf6	0.002494	0.033808	0.658172084	0.892056601
28293	BC042161	19	7651760	7653756	BC022836	TRAPPC5	0.000426	0.01229	0.658481892	0.892056601
7325	THC1581184	3	186483388	186493568	BC036593		0.002943	0.037635	0.667438809	0.893757793
28551	AF161369	19	15245641	15246558	AK024595		0.003032	0.038079	0.668539488	0.893757793
28426	I_1000433	19	11693099	11710810	X51760		0.000838	0.017357	0.668966825	0.893757793
7303	NM_005787	3	185442822	185449479	NM_005787	ALG3	0.003823	0.043572	0.669722048	0.893757793
7265	BC010630	3	184036478	184123517	AB023173	ATP11B	0.000862	0.017499	0.6735876	0.893757793
28141	NM_006339	19	3522659	3530079	NM_006339	HMG20B	0.000213	0.009371	0.674675103	0.893757793
28220	BC009557	19	5629432	5631907	AK091768		0.001968	0.030332	0.678013669	0.894878335
6547	NM_024548	3	102926151	102972095	AK022267	LRRIQ2	0.000708	0.01603	0.682135863	0.895225016
28457	NM_016145	19	12639868	12641449	NM_032332	PTD008	6.60E-05	0.005471	0.683263661	0.895225016
7055	NM_001086	3	153014558	153028979	NM_001086	AADAC	0.000554	0.01459	0.688664883	0.8982978
6949	NM_152282	3	142433353	142496444	NM_152282	ACPL2	0.000278	0.010568	0.696428608	0.8982978
6915	NM_016216	3	139362913	139376513	U10510	DBR1	3.00E-06	0.00208	0.69942205	0.8982978
7245	NM_020409	3	180788959	180805145	BC021575	MRPL47	6.70E-05	0.005471	0.700179485	0.8982978
7185	NM_020390	3	172090923	172090982	NM_020390	EIF5A2	0.002155	0.031376	0.701512401	0.8982978
7244	NM_178042	3	180763410	180788890	NM_004301	ACTL6A	0.000165	0.008545	0.702192966	0.8982978
28244	I_1100823	19	6323444	6326077	NM_032306	ALKBH7	0.001367	0.023135	0.703124462	0.8982978
28555	AK027615	19	15393771	15401599	BC030281	WIZ	0.004367	0.046409	0.715850883	0.905708113
27985	NM_017876	19	598534	614227	NM_017876	RNF126	0.000724	0.01603	0.71594972	0.905708113
28234	NM_003624	19	5867154	5908994	NM_003624	RANBP3	0.001968	0.030332	0.718283525	0.905708113
28053	NM_003926	19	1527678	1543652	NM_003926	MBD3	0.001367	0.023135	0.720543319	0.905708113
7311	NM_144635	3	185540960	185546768	NM_144635	C3orf40	0.003592	0.041652	0.721539053	0.905708113
6559	AF283775	3	108847132	109007394	NM_020235	BBX	0.003032	0.038079	0.725292828	0.906159338
28497	NM_032285	19	13736345	13746104	NM_032285	MGC3207	0.002124	0.031376	0.726946767	0.906159338
7278	NM_017644	3	184836113	184879958	AK024270	KLHL24	0.000256	0.010153	0.741412601	0.920993508
7259	BC041898	3	182764010	182941337	BC013923	SOX2OT	0.003303	0.039837	0.746082201	0.922382127
28370	NM_080665	19	10277099	10287686	NM_080665	MGC19604	0.00143	0.023796	0.747669078	0.922382127
28174	NM_018074	19	4198068	4220087	BC007206	FLJ10374	0.001367	0.023135	0.752041057	0.923263661
28123	BC001528	19	3073372	3074994	NM_152294	GNA11	0.002802	0.036211	0.755140281	0.923263661
28164	I_966199	19	3989414	3990383	NM_015897	PIAS4	0.000324	0.010812	0.756098932	0.923263661
7267	BC033880	3	184097183	184115093	AB023173	ATP11B	0.000221	0.009371	0.760606395	0.925619308
28038	I_3549648	19	1389379	1391492	NM_170711	DAZAP1	0.001367	0.023135	0.767227198	0.927380864
28275	THC1468550	19	7038155	7039455	NM_024341	ZNF557	0.002217	0.032106	0.772467016	0.927380864
6895	AK023738	3	135687331	135776551	NM_025180	CEP63	0.003094	0.038079	0.772963659	0.927380864

7255	THC1453312	3	182170914	182172048	AF075028		0.000143	0.008182	0.774602612	0.927380864
7258	NM_145261	3	182190128	182190187	NM_145261	DNAJC19	8.00E-06	0.002995	0.774970082	0.927380864
28235	NM_007321	19	5871914	5884670	NM_003624	RANBP3	0.000724	0.01603	0.789025936	0.940084182
30697	NM_022105	20	61006800	61039898	NM_022105	DIDO1	0.002619	0.034848	0.790822906	0.940084182
28054	NM_006830	19	1548205	1556488	NM_006830	UQCR	0.002494	0.033808	0.800204559	0.948097151
7264	ENST00000305540	3	184036478	184123517	AB023173	ATP11B	0.000664	0.015855	0.80309694	0.948394084
28179	NM_032868	19	4294556	4311082	NM_003025	FLJ14981	0.003941	0.043729	0.807325623	0.950261963
30683	ENST00000317311	20	60396105	60397157	AK057740	RPS21	0.000165	0.008545	0.815600651	0.954904286
6928	NM_006219	3	139856229	140036429	Z36836	PIK3CB	0.003094	0.038079	0.817371926	0.954904286
28029	NM_024407	19	1334856	1346602	AK094875	NDUFS7	0.000324	0.010812	0.821223978	0.954904286
28064	I_1854903	19	1825877	1827195	BM547883		0.002802	0.036211	0.8256912	0.954904286
28589	NM_006387	19	16489687	16514276	NM_006387	CHERP	0.001072	0.020134	0.83115867	0.954904286
28391	NM_006858	19	10804118	10807993	NM_006858	TMED1	0.002103	0.031336	0.833676487	0.954904286
7450	I_3589590	3	196924489	196925549	AW138772		0.000335	0.010953	0.833798985	0.954904286
7195	AK091897	3	172853734	172878366	NM_002662		0.002053	0.03123	0.834142894	0.954904286
28233	NM_007322	19	5867145	5929157	NM_003624	RANBP3	0.000938	0.018362	0.835208762	0.954904286
28065	NM_080924	19	1827979	1834331	NM_031213	FAM108A1	0.002494	0.033808	0.843756024	0.955579199
7233	NM_171828	3	180443296	180467540	NM_006218	KCNMB3	0.003303	0.039837	0.844283113	0.955579199
6918	AL096748	3	139465158	139499936	NM_015396	ARMC8	0.000655	0.015855	0.850591388	0.955579199
6899	L07590	3	137167221	137348090	NM_002718	PPP2R3A	7.00E-05	0.005471	0.85153781	0.955579199
7243	AF285120	3	180763378	180803431	NM_004301		0.000981	0.018905	0.852709641	0.955579199
7284	AK090720	3	185004474	185007080	NM_018023		0.003143	0.038293	0.85527049	0.955579199
28475	NM_013976	19	12863083	12872131	NM_000159	GCDH	0.000359	0.011452	0.857121672	0.955579199
28421	NM_016581	19	11477732	11500983	NM_016581	SITPEC	0.00031	0.010812	0.862187438	0.955579199
6927	NM_018147	3	139834562	139834621	NM_018147	FAIM	8.00E-05	0.00577	0.862348719	0.955579199
6950	AK092355	3	142525754	142646691	NM_024724	ZBTB38	0.003488	0.040969	0.866914545	0.955579199
28593	I_961604	19	16544435	16600042	AK026694		0.000187	0.008665	0.867357701	0.955579199
7172	AK023029	3	171349418	171382218	AK022455	PHC3	0.001367	0.023135	0.872921823	0.955579199
7238	I_943905	3	180593215	180595063	NM_021629	GNB4	0.000293	0.010736	0.878383661	0.955579199
7247	NM_002492	3	180805277	180825019	NM_002492	NDUFB5	7.90E-05	0.00577	0.880119691	0.955579199
28108	NM_003021	19	2705669	2734354	NM_003021	SGTA	0.000324	0.010812	0.883494869	0.955579199
28452	I_1000366	19	12600812	12602721	NM_153358	FLJ90396	0.000174	0.008665	0.88556515	0.955579199
28134	NM_016263	19	3457304	3487194	BG029787	FZR1	0.004404	0.046409	0.885684305	0.955579199
7254	X90874	3	182113154	182177035	AF075028	FXR1	0.000255	0.010153	0.890081919	0.955579199
28170	NM_016539	19	4125104	4133603	NM_016539	SIRT6	0.000282	0.010586	0.8902358	0.955579199
7353	NM_001622	3	187813556	187821820	NM_001622	AHSG	0.004144	0.045148	0.89521351	0.955579199
28095	AF152351	19	2377843	2378589	NM_012458		0.003522	0.040969	0.897760887	0.955579199
7287	NM_018622	3	185029864	185085402	NM_018622	PARL	0.000138	0.008153	0.898227292	0.955579199
28120	AK095154	19	3003690	3008126	NM_001130	AES	0.004916	0.049385	0.898286963	0.955579199
28008	NM_019112	19	991116	1016576	NM_019112	ABCA7	0.002494	0.033808	0.899681809	0.955579199
28546	I_3549137	19	15131434	15172794	NM_000435	NOTCH3	0.004917	0.049385	0.912129757	0.965942722
28316	NM_005001	19	8282195	8292290	NM_005001	NDUFA7	0.000143	0.008182	0.91970768	0.96953262
28363	NM_003755	19	10086699	10086758	NM_003755	EIF3S4	0.000617	0.015566	0.920920956	0.96953262
7280	AK024270	3	184882123	184885009	AK024270	KLHL24	0.002599	0.034848	0.931935345	0.975379764
28249	NM_002096	19	6330574	6344384	NM_004158	GTF2F1	0.003522	0.040969	0.93457854	0.975379764
28481	NM_052850	19	12925968	12929050	M77024	GADD45GIP1	0.000552	0.01459	0.936541851	0.975379764
16406	AK090416	9	134443382	134519796	NM_002957		0.004585	0.047788	0.93734267	0.975379764
28615	NM_023937	19	17277477	17278659	NM_023937	MRPL34	0.000814	0.017268	0.941774793	0.977159395
7263	AB023173	3	184036478	184123517	AB023173	ATP11B	0.000255	0.010153	0.945679802	0.978383426
7291	I_3562684	3	185184243	185218406	NM_005688	ABCC5	0.00016	0.008545	0.949708475	0.979727996
28066	NM_031213	19	1827993	1836432	NM_031213	FAM108A1	0.000324	0.010812	0.956407735	0.983811968
28524	NM_138998	19	14380633	14391171	NM_078481	DDX39	0.000502	0.013521	0.963006587	0.985861042
6863	BC007460	3	133518949	133569847	NM_001099	ACPP	0.003094	0.038079	0.969219754	0.985861042
28451	NM_153358	19	12582732	12601677	NM_153358	FLJ90396	0.001867	0.029268	0.970995431	0.985861042
28184	NM_025241	19	4396261	4408790	NM_025241	UBXD1	0.000324	0.010812	0.971614257	0.985861042
7246	A_23_BS425492	3	180789341	180805130	BC021575		0.000387	0.011731	0.974707835	0.985861042
7320	AB018347	3	186033253	186253153	AL833838	KIAA0804	0.001114	0.020501	0.975872398	0.985861042
7266	AF156548	3	184036478	184123517	AB023173	ATP11B	0.000221	0.009371	0.977622649	0.985861042
7236	NM_033540	3	180548177	180593786	NM_021629	MFN1	0.003303	0.039837	0.984502556	0.990017977
24784	BM909113	16	29778011	29786782	BM909113		0.004917	0.049385	0.987360904	0.990118896
15157	AF289600	8	145496556	145498365	AF289600		0.003412	0.04088	0.992574521	0.992574521
